# Combination of interleukin-15 and fractionated radiotherapy for cancer treatment

**DOI:** 10.1186/2051-1426-2-S3-P157

**Published:** 2014-11-06

**Authors:** Joseph Aryankalayil, Karsten Pilones, Ralph Vatner, Sandra Demaria

**Affiliations:** 1 New York University, New York, NY, USA; 2 NYU School of Medicine, New York, NY, USA

## Background

Radiotherapy (RT) can induce T cell-mediated anti-tumor immune responses by multiple mechanisms. However, due to the strongly immunosuppressive tumor microenvironment that hinders T cell function, these responses are ineffective and tumors usually escape. The common gamma-chain cytokines interleukin ( IL)-2 and IL-15 promote the proliferation of activated T cells and, therefore, are prime agents for immunotherapy strategies aimed at sustaining anti-tumor T cell responses. IL-2 at high doses is effective at inducing responses in a subset of cancer patients. However, the benefits of IL-2 are partially offset by serious toxicity and by regulatory T cell (Treg) stimulation. IL-15, on the other hand, has shown low toxicity and lacks Treg stimulatory activity, making it an attractive candidate for testing in combination with RT. Here we tested the hypothesis that IL-15 administration strengthens the pro-immunogenic effect of hypofractionated RT and contributes to inducing effective anti-tumor responses.

## Methods

The poorly immunogenic TSA mouse breast cancer cells were implanted s.c. in syngeneic BALB/c mice on day 0. When tumors became palpable, mice were randomly assigned to one of 4 treatment groups (N = 4-5mice/group): control, RT, IL-15 or RT+IL-15. RT was delivered exclusively to the primary tumor in 8 Gy fractions on days 13, 14 and 15. IL-15 (5 μg/mouse) was administered i.p. or s.c. peritumorally daily for 10 days starting on day 12. Mice were followed for tumor growth or regression. In a separate experiment, mice were euthanized on day 22 to characterize tumor-infiltrating lymphocytes (TILs).

## Results

Tumor growth delay was seen when IL-15 was given peritumorally but not i.p. (p < 0.05 compared to control). Improved tumor regression was seen when RT was combined with IL-15 (p < 0.05), suggesting that IL-15 can potentiate T cell responses elicited by RT. Consistently, analysis of TILs showed a marked increase in CD8+ T cells expressing the activation marker CD137 (35.3% in RT+IL-15 versus 5.90% In control, p < 0.05) while the increase was modest with each monotherapy (18.8% in RT, 20.7% in IL-15, p < 0.05 compared to control). In addition, we found a significant increase in the ratio of effector CD4+ T-cells to Tregs (2.5 in RT+IL-15 versus 0.78 in control, p < 0.05) whereas monotherapy had no effect (1.14 in RT, 0.96 in IL-15).

## Conclusions

Overall these results support the hypothesis that IL-15 is a valuable partner for combination treatments with local RT. We are currently elucidating the mechanisms involved in pre-clinical models in preparation for future testing in patients.

